# Sehschärfenminderung bei prominenter Makula und Myopie

**DOI:** 10.1007/s00347-020-01236-w

**Published:** 2020-09-28

**Authors:** Anna Maria Riedel, Chris P. Lohmann, Michael Ulbig

**Affiliations:** Klinik und Poliklinik für Augenheilkunde, Klinikum rechts der Isar der Technischen Universität München, München, Deutschland

## Anamnese

Im April 2017 stellte sich eine 53-jährige kurzsichtige Patientin wegen beidseitiger Sehschärfenminderung in unserer Sprechstunde vor. Die Patientin beklagte einen Visusabfall, links mehr als rechts, seit etwa 2 Monaten mit Wahrnehmung von Metamorphopsien links. Versorgt mit formstabilen Kontaktlinsen, betrug die Myopie rechts −6,75 dpt und links −7,25 dpt.

## Klinische Befunde

Die Sehschärfe mit Kontaktlinse betrug am rechten Auge 0,3 und am linken Auge 0,4 pp. Die Untersuchung der vorderen Augenabschnitte zeigte eine geringgradige Linsentrübung. Der Augeninnendruck betrug beidseits 13 mm Hg. Bei der Fundoskopie zeigten sich beidseits ein Conus myopicus, eine aufgelockerte Makula mit Pigmentepithelverschiebungen sowie gestreckt verlaufende Gefäße.

## Retinale Bildgebung

**Figure 1:**
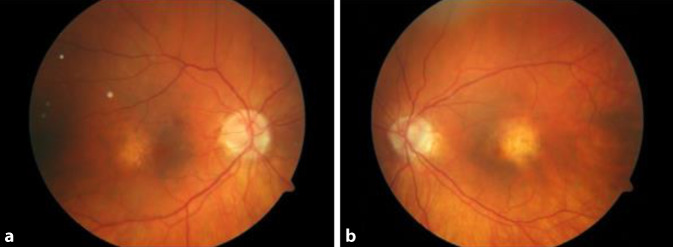

**Figure 2:**
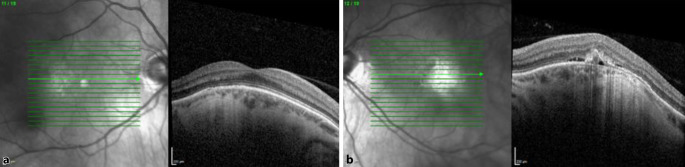

Die optische Kohärenztomographie (OCT) (HRA und OCT-Spectralis; Heidelberg Engineering GmbH, Heidelberg, Deutschland) zeigte am rechten Auge einen foveal regelrechten Strukturaufbau mit parafovealen Pigmentepithelverschiebungen (Abb. 1). Auch am linken Auge fanden sich Pigmentepithelverschiebungen und zusätzlich der Nachweis subretinaler Flüssigkeit (Abb. 2). Die Höhe der makulären Prominenz betrug am rechten Auge 575 µm und am linken Auge 576 µm.

**Figure 3:**
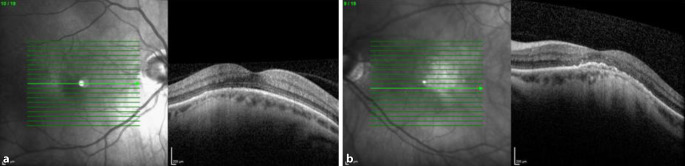

In der Fluoreszenzangiographie (FLA) präsentierte sich am rechten Auge eine Hyperfluoreszenz im Bereich der parafovealen Läsionen. Das linke Auge wies eine teils verstärkte sowie teils reduzierte Autofluoreszenz bei Pigmentepitheldefekten auf. In der Frühphase der FLA wurden eine parafoveale Hyperfluoreszenz mit Staining und im weiteren Verlauf das Vorhandensein einer dezenten Leckage diagnostiziert (Abb. 3 und 4). Die Leckage wurde als Hinweis auf eine chorioidale Neovaskularisation (CNV) bewertet.

**Figure 4:**
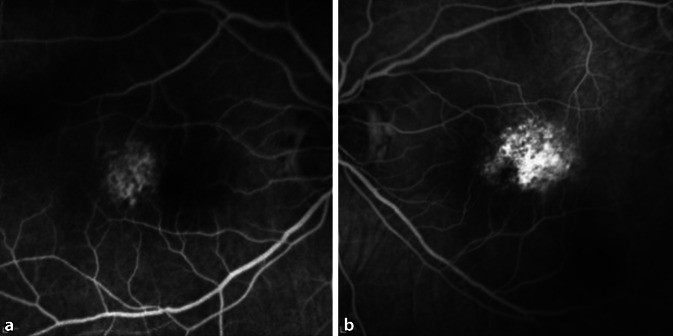

## Wie lautet Ihre Diagnose?

## Therapie und Verlauf

Die initiale Therapie umfasste die 3‑malige intravitreale Medikamenteneingabe von Ranibizumab am linken Auge jeweils im Abstand von 4 Wochen. Die Ansammlung subretinaler Flüssigkeit war im Verlauf rückläufig, jedoch nicht vollständig aufgehoben, sodass die Therapie mit weiteren intravitrealen Injektionen von Ranibizumab fortgesetzt wurde. Die Patientin erhielt im 4‑wöchigen Abstand insgesamt 8 Injektionen, wodurch sich die subretinale Flüssigkeit vollständig resorbierte. Bei einer Kontrolle nach 1 Jahr wurde erneut subretinale Flüssigkeit links nachgewiesen, sodass die intravitreale Medikamenteneingabe mit Ranibizumab nach dem Treat & Extend-Schema erneut aufgenommen wurde.

## Diskussion

Die Dome-shaped-Makulopathie zeichnet sich durch eine makuläre Prominenz aus und geht oft mit einer Sehschärfenminderung sowie der Wahrnehmung von Metamorphopsien einher. Sie ist häufig assoziiert mit einer höheren Myopie sowie einem Staphyloma posticum, wobei Autoren wie Lorenzo et al. darauf hinwiesen, dass eine hohe Myopie keine zwingende Voraussetzung für eine Dome-shaped-Makulopathie ist [1]. Es gibt Hinweise darauf, dass eine Ablösung des retinalen Pigmentepithels sowie eine extrafoveale Retinoschisis häufiger vorkommen können. Die genaue Pathogenese für diese Befunde konnte bisher nicht eindeutig geklärt werden. Gaucher et al. nehmen an, dass die makuläre Vorwölbung durch eine Verdickung der Aderhaut entsteht [2]. Diskutiert wurde jedoch auch ein Defekt in der Bruch-Membran, der einen Kollaps der hinteren Netzhautschichten verursacht sowie eine vitreomakuläre Traktion, die für eine Vorwölbung der Makula verantwortlich sein könnte. Andere Autoren wie Imamura et al. vermuten, dass eine Verdickung der Sklera ursächlich sein kann [3]. Hierdurch könnte die Choroidea komprimiert werden, sodass die Blutzirkulation im Bereich der hier vorhandenen Gefäße gestört ist und ein Flüssigkeitsaustritt entsteht. Viola et al. diskutierten, dass eine Verdickung der Aderhaut für die makuläre Vorwölbung ursächlich sein kann, und vergleichen den Pathomechanismus mit dem der Chorioretinopathia centralis serosa (CCS) [4]. Differenzialdiagnostisch muss bei Visusminderung, Metamorphopsien und der Ansammlung subretinaler Flüssigkeit, wie im hier beschriebenen Fall, eine Chorioretinopathia centralis serosa und auch eine sekundäre CNV in Erwägung gezogen werden. Hinsichtlich dieser beiden Veränderungen sollten engmaschige Nachuntersuchungen durchgeführt werden.

Der Verlauf der Dome-shaped-Makulopathie ist meist über viele Jahre stabil. Die Krankheitsmerkmale und Symptome bei der Dome-shaped-Makulopathie treten häufig erst in der 2. Lebenshälfte auf. Typische Risikofaktoren einer CCS wie Stress und die Einnahme von Steroiden, ein geringes Alter oder das männliche Geschlecht sind nicht typisch für eine Dome-shaped-Makulopathie.

**Diagnose:** Dome-shaped-Makulopathie beidseits mit sekundärer CNV links

Therapeutisch konnte bisher für die Dome-shaped-Makulopathie kein allgemein akzeptiertes Konzept erarbeitet werden. Die intravitreale Injektion von VEGF-Hemmern konnte einen oft nur vorübergehenden Visusanstieg zeigen, ist aber insbesondere bei Hinweisen auf eine sekundäre CNV zu empfehlen. Die intravitreale Eingabe von Ranibizumab führte bei unserer Patientin zu keinem anhaltenden Erfolg und wurde in ein Treat & Extend-Schema überführt [1].

Die orale Einnahme des Aldosteronantagonisten Spironolacton zeigte in der Studie von Dirani et al. bei einigen Patienten eine Reduzierung der subretinalen Flüssigkeitsansammlung. Sie führte bisher aber nicht zu einer langfristigen Befundverbesserung [5]. Therapeutisch ebenfalls zu erwägen ist die photodynamische Therapie, die durch intravenöse Gabe des photosensiblen Medikaments Verteporfin und anschließend gezielter nichtthermischer Laserapplikation die Endothelzellen der Aderhaut schädigt. In der darauf folgenden Abheilung erlangen die Gefäße dann ihre normale Permeabilität („vascular remodeling“).

## Fazit für die Praxis

Die Dome-shaped-Makulopathie tritt häufig bei Patienten mit einer Myopie in der zweiten Lebenshälfte auf. Differenzialdiagnostisch sollte bei der Ansammlung subretinaler Flüssigkeit sowie bei der Abhebung des retinalen Pigmentepithels bei der Dome-shaped-Makulopathie auch an eine sekundäre CNV und an eine Chorioretinopathia centralis serosa gedacht werden. Eine myope CNV ist bei fehlender Prominenz der Makula zu erwägen. Die Nachuntersuchungsintervalle sollten entsprechend gestaltet werden. Therapeutisch können die intravitreale Medikamenteneingabe von VEGF-Hemmern, insbesondere bei Vorliegen einer sekundären CNV, und alternativ auch die photodynamische Therapie bei der Dome-shaped-Makulopathie erwogen werden.
